# New Method for Enhancing Coconut (*Cocos nucifera* L.) Embryo Dehydration: An Important Step Towards Proficient Cryopreservation

**DOI:** 10.3390/plants14040600

**Published:** 2025-02-17

**Authors:** Amirhossein Bazrafshan, Sisunandar Sudarma, Sundaravelpandian Kalaipandian, Julianne M. Biddle, Zhihua Mu, Eveline Yee Yan Kong, Naga Prafulla Chandrika Nulu, Steve W. Adkins

**Affiliations:** 1 School of Agriculture and Food Sustainability, The University of Queensland, Gatton, QLD 4343, Australia; s.kalaipandian@uq.edu.au (S.K.); julianne.biddle@uq.edu.au (J.M.B.); zhihua.mu@uqconnect.edu.au (Z.M.); e.kong@uq.edu.au (E.Y.Y.K.); s.adkins@uq.edu.au (S.W.A.); 2 Department of Biology, Universitas Muhammadiya Purwokerto, Banyumas 53182, CJ, Indonesia; sisunandar@ump.ac.id; 3 Centre for Horticultural Science, Queensland Alliance for Agriculture and Food Innovation, The University of Queensland, Indooroopilly, QLD 4068, Australia; 4 Department of Bioengineering, Saveetha Institute of Medical and Technical Sciences (SIMATS), Saveetha School of Engineering, Chennai 602105, TN, India; 5 School of Breeding and Multiplication Sanya Institute of Breeding and Multiplication, Hainan University, Sanya 572025, China

**Keywords:** coconut conservation, moisture removal, embryo desiccation, cryopreservation, sucrose, pre-treatment, high-recovery, embryo size

## Abstract

The coconut (*Cocos nucifera* L.), a valuable tropical crop, is rapidly declining in genetic diversity due to natural disasters, pest and disease attack, and land clearing for other crops. Seed banking is impractical for coconut conservation due to its large, recalcitrant seed, and maintaining field gene bank collections is costly and vulnerable to environmental pressures. Cryopreservation offers a promising alternative method for conserving coconut genetic diversity, but the success in recovering cryopreserved materials remains limited, with few studies consistently reporting high rates of recovery. This highlights the need for improved cryopreservation protocols, particularly in tissue dehydration, which is one of the critical steps in the process of cryopreservation and plant recovery. A desiccator was developed that enabled rapid embryo dehydration with ultra-dry airflow. The desiccator reduced embryo moisture content to 20% (the predetermined viability threshold) within 6 h representing a 2-h improvement when compared to a previous dehydration approach, while maintaining a high germination rate (71%). Smaller embryos (500 to 550 mg fresh weight) desiccated faster than larger embryos (800 to 900 mg fresh weight) but germination was reduced (30%), making small embryos unsuitable for cryopreservation. A 5-day sucrose (0.4 M) pre-treatment further reduced the dehydration time to 4 h, while maintaining a high germination rate (70%). These advances in the use of a sucrose pre-treatment, the rapid embryo dehydration, and selection of large embryos size will help to enhance the success of coconut embryo cryopreservation and recovery.

## 1. Introduction

The coconut palm (*Cocos nucifera* L.) is extensively cultivated across many tropical and subtropical regions of the world, sustaining the livelihoods of many millions of smallholder farmers [1]. At present, the crop is cultivated on 12 million ha of land in over 93 countries with an annual production of ca. 62 million t of dehusked fruit [2,3]. A significant portion of the existing coconut plantations, established over 70 years ago, are now exhibiting reduced productivity [4] due to palm senility, and the prevalence of severe diseases such as lethal yellowing [5] and cadang-cadang [6], pest invasions such as those caused by rhinoceros beetle (*Oryctes rhinoceros* L.), the red palm weevil (*Rhynchophorus ferrugineus* Olivier), and the coconut black-headed caterpillar *Opisina arenosella* Walker; [7]. Consequently, these senile and affected palms are being systematically destroyed in some regions, and the land repurposed for alternative crops or other uses. However, this transition away from traditional coconut-production carries a profound consequence, and that is the loss of locally adapted germplasm. Such an event does not only exacerbate the challenges faced by coconut breeders but also highlights the urgent need for a functional approach to coconut conservation to sustain its genetic diversity. The widely used seed banking method for orthodox crops cannot be used for conserving coconut germplasm due to the desiccation-sensitivity or recalcitrant nature and very large size of the coconut seed [8].

Traditionally, field gene banks have been used as the primary method for conserving coconut germplasm [9]. This approach has been widely employed within the agricultural community, but it faces significant challenges as it involves managing large areas of land and a high exposure to pests, diseases and environmental threats. An alternative approach for the ex-situ conservation of coconut germplasm is cryopreservation, which utilises in vitro methods that involve a process of tissue dehydration followed by ultra-cold storage of the dehydrated tissue. Cryopreservation of coconut germplasm has proven to be quite challenging. Several researchers have experimented with different types of explants such as for coconut germplasm preservation and were not successful to achieve high success rate. To date, most reliable and higher success rate for coconut germplasm cryopreservation has been achieved using mature coconut embryos as explants [10].

In many plant species, the success of cryopreservation depends on the moisture content of the tissues prior to cryopreservation [11] and the rate at which moisture is removed from these tissues [10,11,12,13]. Although advanced cryopreservation methods like the vitrification techniques have been recently developed, the physical dehydration techniques still remain a valuable tool in the cryopreservation strategies of species that produce large numbers of zygotic embryos, but no vegetative organs. The historical effectiveness, simplicity, and accessibility of zygotic embryos justify their selection, particularly for recalcitrant species and in situations where the advanced methods are not feasible [14,15].

A rapid, physical dehydration technique using a silica gel desiccator has been developed and used for drying coconut embryos prior to cryopreservation. This past approach could dehydrate embryos to the viability threshold level of 20% fresh weight (FW) in 8 h, with 40% recovery of normal plants to soil after cryopreservation [10]. The germinability of embryos after recovery from cryopreservation significantly decreased when the moisture content of the embryos was either below or above the 20% threshold level [10]. However, to improve further the recovery of plants to soil it is important to reduce further the duration time necessary for rapid physical dehydration. To meet these requirements, a new desiccator was designed, built, and tested based on the earlier prototype developed by Sisunandar et al. [10].

Apart from a rapid dehydration step, a sucrose pre-treatment step applied to the tissues prior to cryopreservation has been shown to improve tissue recovery [16]. Such pre-treatments are thought to (a) enhance tissue survival through the cryopreservation process and (b) to speed up the time to undertake tissue dehydration. For instance, pre-treating tissues in sucrose solutions helped prevent ice formation within cells during the freezing process, thereby enhancing plant recovery following cryopreservation process for several species [17,18,19]. It has also been suggested that a sucrose pre-treatment can expedite the dehydration process by facilitating water absorption from the tissues through osmotic action [20,21].

In our current study, we focused on optimizing the dehydration process for cryopreservation of coconut embryos. The objectives of this study were to (a) trial a newly designed desiccator for its ability to rapidly, physical dehydrate coconut embryos to their viability threshold, and how this dehydration treatment would affect the ability of the embryos to germinate, (b) to assess the impact of the rapid dehydration technique on the germination of either small or large embryos and (c) to examine the impact of various sucrose pre-treatments on the time of dehydration time and germination of embryos.

## 2. Results

### 2.1. Rapid, Physical Dehydration of Coconut Embryos (Experiment 1)

**Figure 1:**
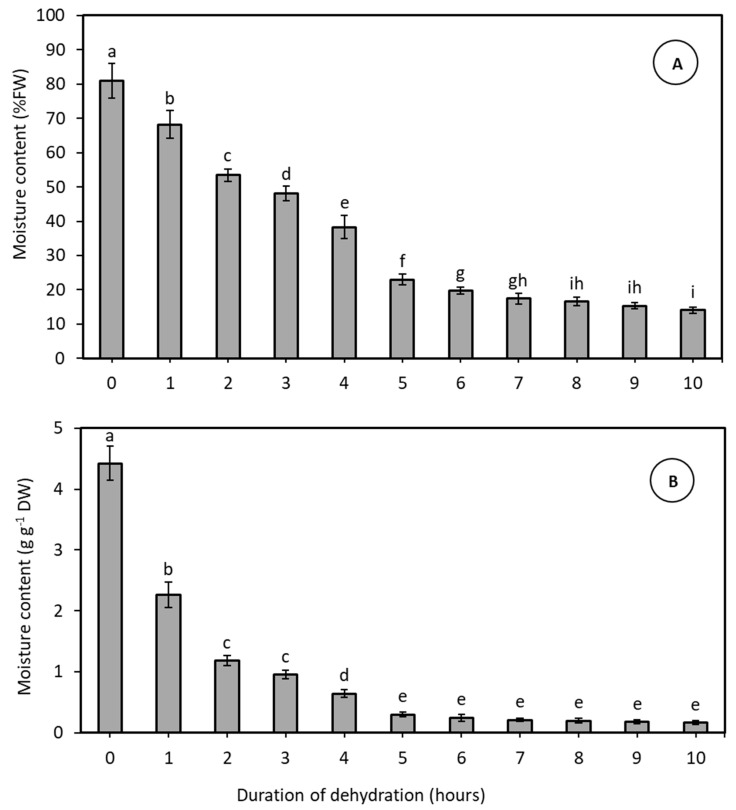
The mean moisture content of zygotic embryos of varieties WAT and MYD, either expressed as (**A**) a percentage of tissue fresh weight or as (**B**) moisture content per g dry weight, after various times of dehydration using the rapid, physical dehydration desiccator. The data at each time point is a pooled value for 20 embryos, 10 from each variety. Error bars represent standard errors of the mean. Letters above the bars indicates a significant difference between treatments.

In this Experiment two varieties (MYD and WAT) were used to determine the dehydration curve required to reduce the embryo moisture content to the 20% threshold level. As the rate of drying did not significantly differ between the two varieties utilised in this study, the data from both varieties were pooled for further analysis. The rate of rapid, physical dehydration comprised of two distinct phases (Figure 1A,B). In the initial 5-h phase of dehydration, there was a substantial decline in embryo moisture content from 81% FW (or 4.42 g g^−1^ DW) to 23% FW (or 0.30 g g^−1^ DW). Subsequently, from 6 to 10 h, a further gradual reduction in embryo moisture content occurred, reaching ca. 14% FW (or 0.16 g g^−1^ DW). The critical moisture threshold of 20% (or 0.25 g g^−1^ DW) was achieved after 6 h of drying (Figure 1A), and therefore 6 h of dehydration was determined to be the appropriate physical dehydration time for these two varieties.

### 2.2. Embryo Viability After Physical Dehydration (Experiment 2)

**Figure 2:**
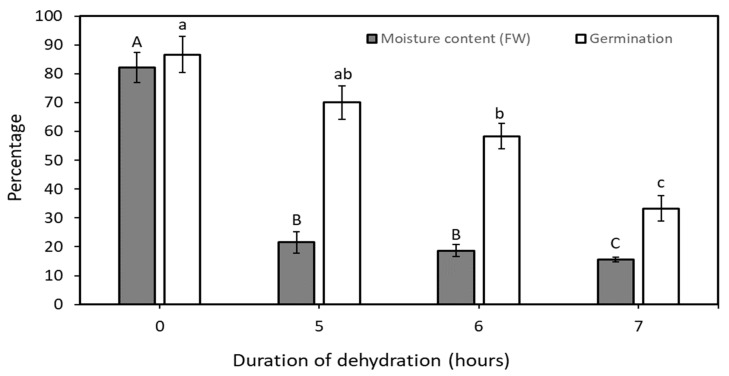
The percentage moisture content and viability of coconut embryos (CAIRNS) determined after various periods of dehydration using the new rapid, physical dehydration desiccator. The moisture content data was determined from 10 embryos, while the viability data (germination percentage) is for three replications of 20 embryos and for four dehydration times (0, 5, 6 and 7 h). Error bars represent the standard error of the mean. Letters above the bars indicates a significant difference within that data set.

In this Experiment one variety (CAIRNS) was used to determine embryo viability decline during the rapid, physical dehydration process required to reduce the embryo moisture content to the 20% threshold level. The rate of dehydration of CAIRNS embryos was similar to that of WAT and MYD but not identical, reaching the critical moisture threshold of 20% after 5 h. The viability of these embryos was 70%, showing no significant difference compared to non-dehydrated embryos (85%; Figure 2). However, the percentage of viable embryos declined to 60% after further dehydration for 6 h (moisture content of 18% FW), followed by a sharp decrease to 30% after 7 h of rapid dehydration (moisture content of 12% FW; Figure 2). Considering the embryo moisture content and the viability result, 5 h of rapid, physical dehydration was the appropriate physical dehydration time for CAIRNS embryos.

### 2.3. Embryo Size on Germinability After Physical Dehydration (Experiment 3)

**Figure 3:**
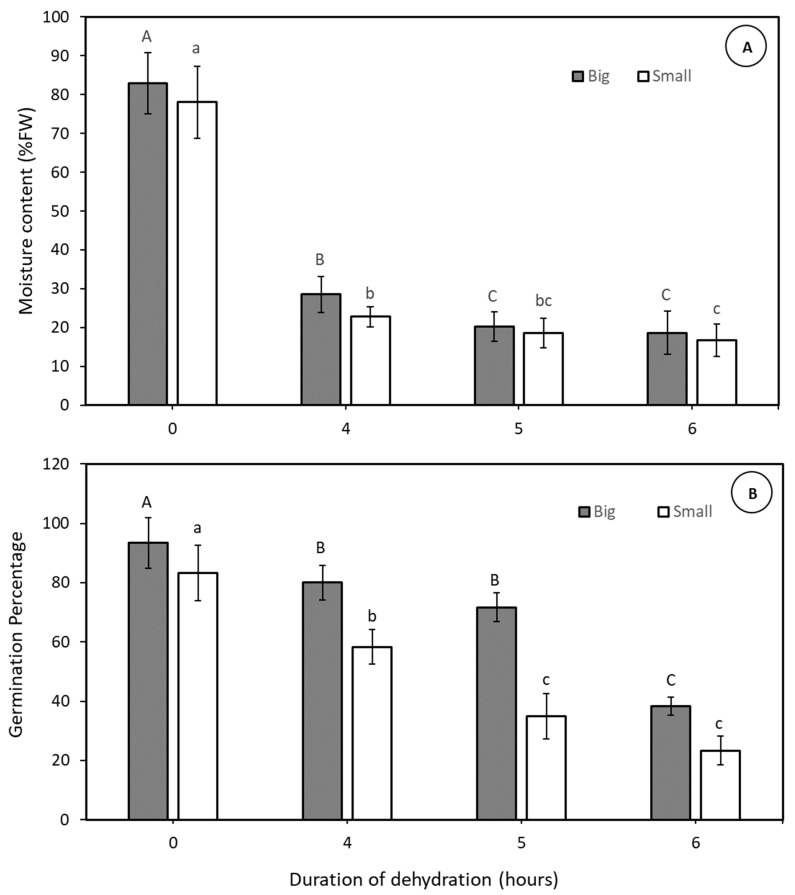
The percentage moisture content (**A**) and the subsequent viability percentage (**B**) of coconut embryos (CAIRNS) after various periods of dehydration using the rapid, physical dehydration desiccator. The embryos had been classified as either being large (800 to 900 mg FT) or small (500 to 550 mg FW) for that variety The moisture content data is based on 10 replicate embryos, while the germination percentage data is from three replications of 20 embryos; Error bars represent the standard error of the mean. The letter above each bar indicates a significant difference within data sets.

In this Experiment one variety (CAIRNS) was used to determine embryo moisture loss and viability decline during the rapid, physical dehydration process of two classes of embryos, large and small. The moisture loss dynamics from small embryos mirrored that seen in large embryos in Experiment 2, occurring in two phases, albeit at a faster pace for the former (Figure 3A). Within 4 h of rapid physical dehydration, the moisture content in small embryos had decreased from 78 to close to 20% moisture content, a rate quicker than that observed from large embryos, which achieved the same value after 5 h of dehydration. Despite the reduction in moisture content to 20% FW, the viability of small embryos declined to 58% large embryos remained high (71%) post rapid, physical dehydration (Figure 3B). However, extending the dehydration treatment by an additional hour (to 18% FW) resulted in a drop in viability to 38% for small embryos and 48% for large embryos. In conclusion, although small embryos dried more rapidly than large embryos, their viability became unacceptably low (Figure 3B).

### 2.4. Sucrose Pre-Treatments on Tissue Dehydration (Experiment 4)

**Figure 4:**
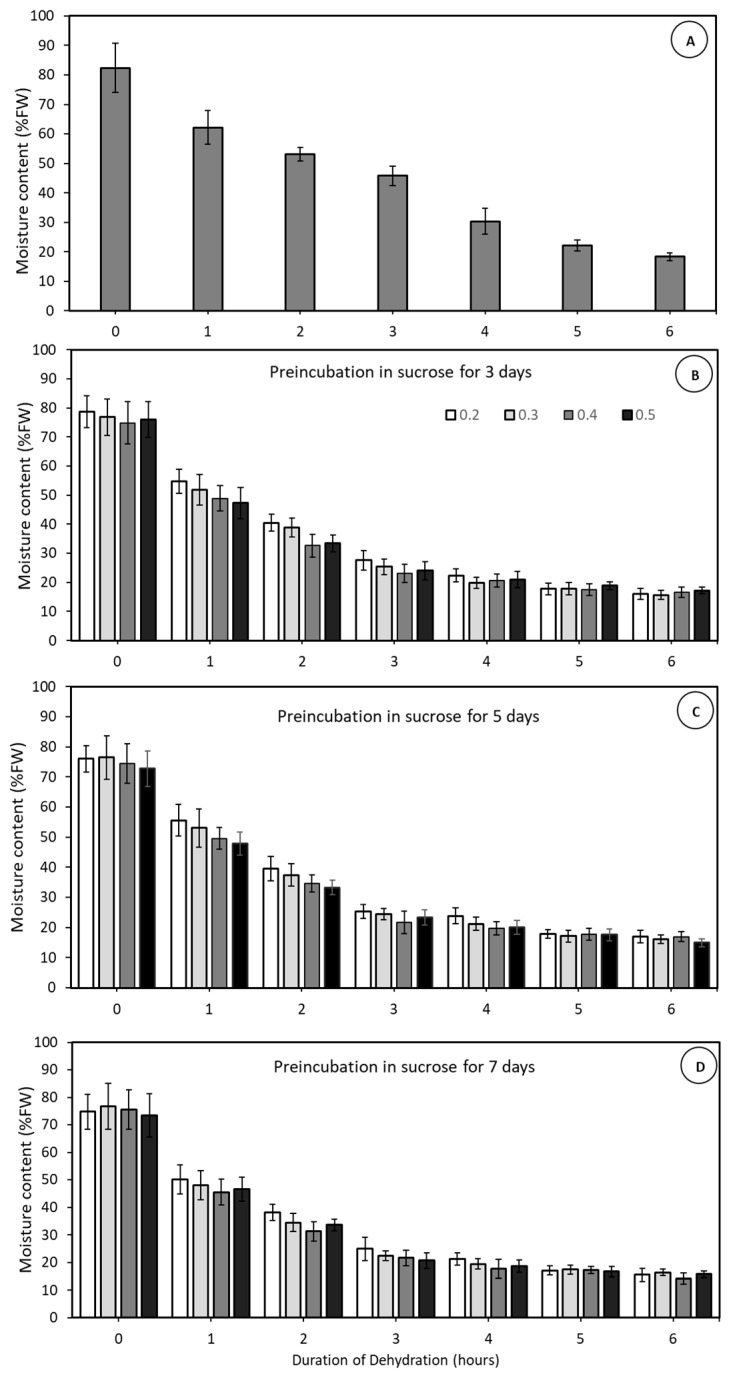
The percentage moisture content of coconut embryos after various times of dehydration using the rapid physical dehydration desiccator. The embryos had been pre-incubated in different concentrations of sucrose (0.0, 0.2, 0.3, 0.4 or 0.5 M) for either 0 (**A**), 3 (**B**), 5 (**C**) or 7 (**D**) days. The data is from 10 replicates each involving a single embryo, Error bars represent the standard error of the mean.

**Figure 5:**
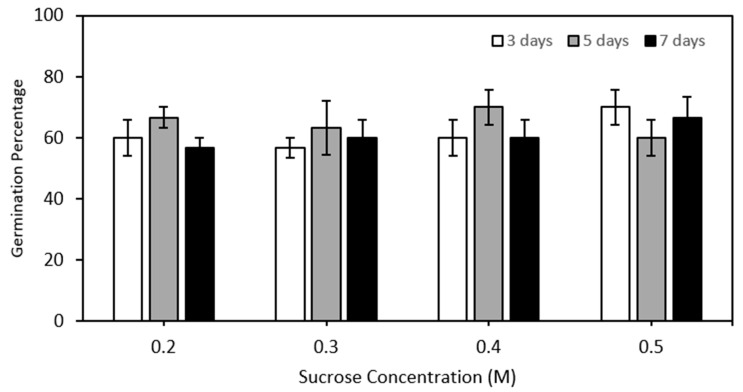
The germination percentage of coconut embryos after 4 h of dehydration using the rapid physical dehydration desiccator. The embryos had been pre-incubated in different concentrations of sucrose (0.0, 0.2, 0.3, 0.4 or 0.5 M) for 3, 5, 7 days. The data is from three replications of 10 embryos; bars represent the standard error of the mean.

This experiment aimed to evaluate the effect of four sucrose pre-treatment concentrations, applied at one of three different times, on the moisture content decline during rapid physical dehydration of large (>700 mg FW) MYD embryos over 7 days. The sucrose pre-treatments exhibited a notable impact on embryo moisture content during rapid, physical dehydration (Figure 4). In the absence of sucrose (Figure 4A), the 20% FW moisture content value was achieved following 6 h of drying. However, when embryos were pre-cultured in either 0.2, 0.3, 0.4, or 0.5 M sucrose solutions, the same moisture content was reached after only 4 h (Figure 4B–D). The time of application (either 3, 5 or 7 days) of sucrose had little effect on embryo moisture decline (Figure 4B–D). Embryos pre-treated with sucrose and subjected to dehydration for 3 to 4 h, achieving a moisture content of approximately 20%, exhibited high viability, with germination rates ranging between 60% and 70% (Figure 5), with no significant differences as compared to embryos dehydrated without a sucrose pre-treatment after 6 h. Hence, a sucrose pre-treatment at any concentration between 0.2 to 0.5 M and applied at any time between 3 to 7 days could speed up embryo dehydration time to just 4 h.

## 3. Discussion

The objectives of this study were to (a) trial a newly designed desiccator for its ability to rapidly, physical dehydrate coconut embryos to their viability threshold (Experiment 1), and how this dehydration treatment would affect the ability of the embryos to germinate (Experiment 2), (b) to assess the impact of the rapid dehydration technique on the germination of either small or large embryos (Experiment 3) and (c) to examine the impact of various sucrose pre-treatments on the time of dehydration time and germination of embryos (Experiment 4).

The newly designed desiccator retained several advantages of the previous model [11] while incorporating key innovative features that enhance its performance and suitability for gene bank use (Figure 1 and Figure 2). Firstly, the new desiccator can more rapidly dehydrate embryos to their viability threshold. Secondly dehydrate ca. 150 embryos in a single run, as compared to the previous model’s capacity of only 20 embryos. Thirdly, the desiccator maintains an airtight environment, reducing the chances of contamination occurring. Fourthly, the central chamber features a high-power fan, significantly boosting dehydration efficiency with an airflow of 1.24 m³ min^−1^, far surpassing the previous model’s capabilities. Finally, the apparatus is constructed from readily available components, all of which (excluding the external electronics) are autoclavable, detachable, and portable. Despite its compact size, the desiccator is capable of dehydrating large numbers of embryos, from several coconut varieties simultaneously, and can be operated efficiently in a confined space, such as a laminar airflow hood.

The newly designed desiccator was able to reduce the embryo moisture content of an equal mix of MYD and WAT embryos to 20% in just 6 h of dehydration (Figure 1), 2 h faster than the previous apparatus [10]. Prior research has highlighted the superiority of rapid, physical dehydration over slow dehydration methods for maintaining viability in recalcitrant seed tissues [10,22]. This superiority stems from the ability of rapid moisture removal from seed tissues, to achieve a lower moisture content, before tissue viability is compromised. Equally, prolonged moisture loss can also negatively impact cell metabolism by disrupting essential metabolic pathways, leading to the breakdown of cellular structures and impaired enzyme activity [23,24].

For reducing the physical dehydration time, CAIRNS variety embryos achieved a moisture content of 20% in ca. 5 h, 1 h faster than the attainment observed for the two Philippine varieties (Figure 2). This disparity could be because of the varieties studied in the two experiments or from disparities in post-harvest and transportation treatments employed to transport the embryos to the laboratory [10 days air freight of isolated embryos (Experiment 1) versus swift 3 days road freight of intact nuts (Experiment 2)]. Previous research on coconut dehydration protocols has highlighted varietal differences, with viability tissue recovery rates ranging from 56 to 93% across four varieties [25]. Moreover, a diverse spectrum of recovery rates (ranging from 20 to 70%) following dehydration have been documented for an alternate cryopreservation protocol applied to seven coconut varieties [26]. Finally, a novel cryopreservation protocol for coconut embryos, based on rapid physical dehydration [10], was tested across 20 varieties and demonstrated a degree of specificity, with varying effectiveness depending on the variety.

Another crucial aspect explored in this study pertained to the impact of embryo size on post rapid, physical dehydration and survival. Findings revealed that large embryos from the CAIRNS variety (Figure 3) exhibited a higher natural viability rate (>90%), than observed with smaller embryos (80%). The smaller embryos studied achieved a 20% moisture content in just 4 h, as compared to 5 h for larger embryos (Figure 3). Despite the expedited moisture loss achieved by smaller embryos, their viability rate post rapid, physical dehydration was notably lower (50%) as compared to the higher rate observed for large embryos (70%). Consequently, although smaller embryos achieved the target moisture content faster, their lower viability suggests a reduced likelihood of favourable recovery rates post cryopreservation. Hence, the preference lies in selecting larger embryos from a variety when establishing a cryopreservation conservation collection for coconut.

The correlation between a sucrose pre-treatment and moisture content after physical dehydration was examined utilising the NYD variety (Figure 4). In the absence of a sucrose pre-treatment necessitated 6 h of rapid physical dehydration to achieve a 20% moisture content (Figure 4), like the Philippine varieties but 1 h longer than the CAIRNS variety (Figure 3). Notably, sucrose concentrations, ranging from 0.2 to 0.5 M exhibited a pronounced ability to help reduce moisture content during the rapid, physical dehydration step. After 4 h of dehydration, embryos pre-cultured in sucrose had a high viability ranging from 60 to 70% (Figure 5). This indicates that sucrose can accelerate the rate of dehydration without affecting viability. Application of a sucrose pre-treatments prior to rapid, physical dehydration has been shown to improve the dehydration process in several palm species previously [27,28,29,30] while retaining tissue viability.

## 4. Materials and Methods

### 4.1. Rapid Dehydration Apparatus

**Figure 6:**
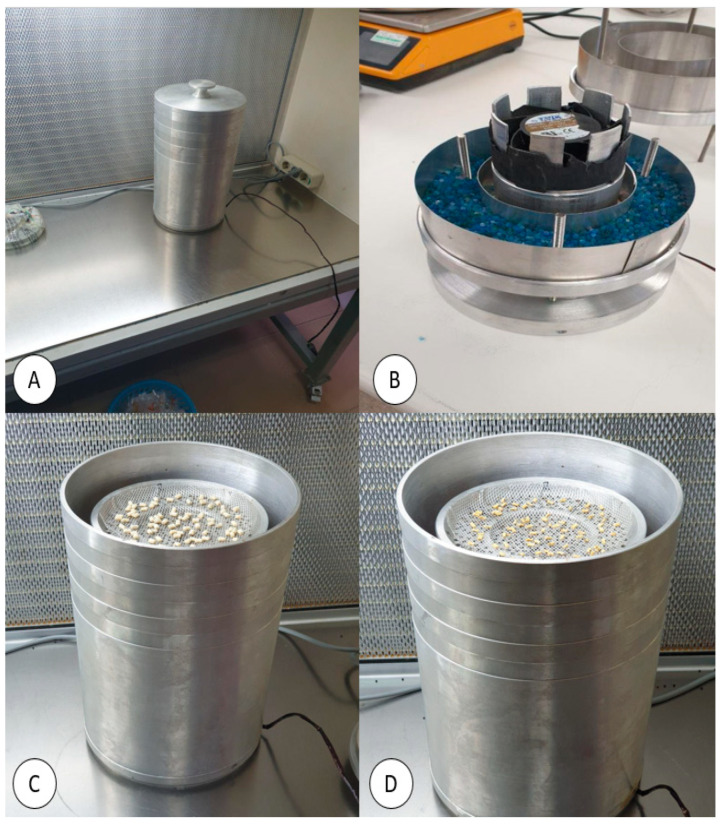
The desiccator used to undertake the rapid, physical dehydration of coconut embryos: (**A**) the intact desiccator; (**B**) an internal view of the desiccator showing one of the sections holding the silica gel and positioned below the fan; (**C**) the desiccator carrying embryos before dehydration, and (**D**) the embryos after dehydration for 4 h.

**Figure 7:**
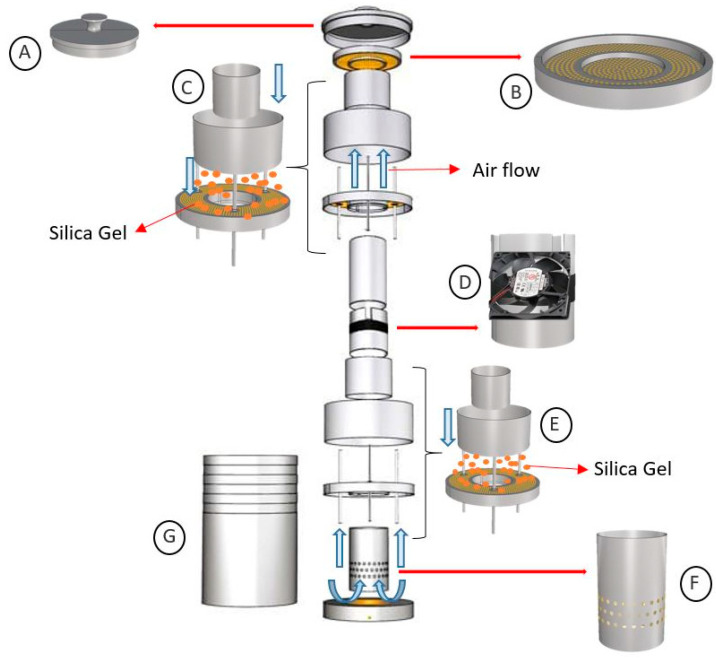
The main body and parts of the desiccator were made from aluminium. At the top of the apparatus there was an airtight lid (**A**) and below this was an aluminium platform, perforated with small ventilation holes, that supported the tissues to be dehydrated (**B**). Circulating ultra-dry air could be passed through the platform driven by a high-power 24 V direct current fan (**D**). The moisture absorbent, silica gel beads were situated in two compartments attached to the central chamber, above and below the fan (**C**,**E**). At the base of the desiccator another chamber carrying large ventilation holes to support air circulation (**F**). A thick (5 mm) aluminium casing (35 × 20 cm; height/diameter) enclosed the system, to prevent contamination and to keep the unit airtight (**G**). Electricity connections to the fan were sealed into the bottom of the encasement.

A rapid, physical dehydration desiccator, adapted from one previously developed [10], was used to dry embryos under sterile conditions. The newly developed desiccator presents several advancements over the previous model, including its lightweight construction, enhanced airflow system, dual moisture absorption chambers, and the ability to process up to 150 embryos per cycle, making it a more efficient and versatile tool for laboratory use (Figure 6). The desiccator body was constructed from aluminium, selected for its lightweight and ability to withstand the high temperatures experienced in the autoclave and the drying oven. An airtight lid was positioned on top of the desiccator, and there was an aluminium platform with small ventilation holes (ca. 1 mm in diameter) at the top layer for placing the samples. These holes facilitated rapid, ultra-dry air movement over the embryos. A cylindrical chamber with aluminium walls was integrated below the platform to serve as the primary conduit for generating airflow. Within this chamber a 24 V direct current axial fan (1.92 W, Omron Corporation, Kyoto, Japan; Figure 7D) was installed, providing an airflow rate of 1.24 m^3^ minute^−1^. To enhance embryo dehydration, silica gel beads (ca. 1 to 3 mm in diameter; Sigma-Aldrich Pty. Ltd., Sydney, NSW, Australia) were placed into a central compartment (5 × 19 cm; height/diameter) above the fan. Another silica gel compartment, located below the fan, served as an additional moisture absorber. The base of the apparatus consisted of another chamber with 3 mm diameter holes in its upper surface to allow for air circulation (Figure 7). To maintain a sterile environment and prevent contamination, a sturdy aluminium case (5 mm thick, with dimensions of 35 × 20 cm; height/diameter) enclosed the entire system in an airtight manner. The desiccator was autoclaved at 121 °C for 20 min and then dried overnight in an oven set at 90 ± 1 °C.

### 4.2. Plant Material Preparation

The coconut varieties studied included West African Tall (WAT), Malayan Yellow Dwarf (MYD), Nias Yellow Dwarf (NYD), and a mix of Dwarf varieties obtained from northern Queensland (referred to as ‘CAIRNS’). For Experiment 1, the zygotic embryos from MYD and WAT were prepared by the Department of Agriculture-Philippine Coconut Authority and imported into Australia under import permit number 0004386518 and placed into a quarantine certified (QC2) laboratory at the University of Queensland (UQ). For Experiment 2 and 3, CAIRNS fruit were freshly harvested, dehusked at the source, and the nuts were transported to UQ by road freight (3 days in transport). For Experiment 4, the zygotic embryos from NYD were prepared and the study performed at the University of Muhammadiyah (UM), Purwokerto, Central Java, Indonesia.

From all varieties, the ca. 11-month-old nuts were split along their circumference using a hammer, and cylinders of the solid endosperm containing the embryo were extracted using a surface-sterilised (70% ethanol; *v*/*v*) 2 cm diameter cork borer. The endosperm cylinders were temporarily stored in a clean glass beaker until further processing, ca. 30 to 60 min later. At that time, the endosperm cylinders were washed with running tap water and rinsed with ethanol (95%; *v*/*v*) to eliminate surface lipids and partially surface sterilise the tissues. In a laminar air flow, the embryos were isolated from the endosperm cylinders using a sterile scalpel blade and forceps, and then transferred into a sterile glass beaker containing sterile water. After ca. 30 min, all embryos of healthy appearance were isolated and surface sterilisation performed by shaking in ethanol (70%; *v*/*v*) for 5 min, followed by immersion in a calcium hypochlorite solution (6%; *w*/*v*) for 10 min with agitation, and finally with two rinses in sterile liquid Y3 medium [31] with sucrose (30 g L^−1^), activated charcoal (2.5 g L^−1^) at pH 5.7. Following rinsing, the embryos were blotted dry on sterile filter paper before being immediately utilised for the next steps in the rapid, physical dehydration experiments.

The embryos imported from the Philippines, were individually placed into 2 mL plastic sterile vials (14 × 48 mm; Thermo Fisher Scientific, Waltham, MA, USA) containing a solid (Gelzan 3 g L^−1^) Y3 medium [31] with sucrose (30 g L^−1^), activated charcoal (2.5 g L^−1^) at pH 5.7 to support and nurture the embryos during transportation to UQ. The vials were packed into Styrofoam boxes and sent to Australia via. an International Courier. In total, ca. 10 days were taken to transport the embryos to UQ, including the days for the quarantine assessment by the staff of the Department of Agriculture, Water, and the Environment. Soon after the embryos were delivered to the QC2 laboratory at UQ, a general assessment was made on their condition in accordance with the import permit of the quarantine materials. High-quality embryos of uniform shape and size (see Section 4.5) were selected for subsequent experimentation. In the quarantine laboratory, all embryos were surface sterilised once again using a sodium hypochlorite solution (0.5%; *v*/*v*) for 3 min, and then rinsed with sterile liquid Y3 medium and immediately used for experiments.

### 4.3. Rapid, Physical Dehydration of Coconut Embryos (Experiment 1)

In this study, batches of 10 MYD and 10 WAT embryos were subjected to rapid dehydration using the desiccator (Figure 7). Dehydration was undertaken, for various periods of time (1 h, 2 h, 3 h, 4 h, 5 h, 6 h, 7 h, 8 h, 9 h, 10 h), in a clean culture room provided with high efficiency purified air and maintained at 25 ± 1 °C. Before starting the dehydration process, the FW of all embryos was determined. The weight was then recorded every hour during the dehydration process in the desiccator. Following the treatments, the batches of embryos were placed into a drying oven set at 90 °C for 24 h to determine their dry weight (DW). Subsequently, themoisture content (MC) of 20 embryos, 10 from each variety (determined from the percent FW and the g g^−1^ DW) at the time of removal from the desiccator and during the hourly occasions leading up to that final time were calculated using the following formulas:

$$\mathrm{MC}\;(\%\;\mathrm{FW})=\frac{FW-DW}{FW}\times 100$$

where the MC was determined from the following calculation:

$$\mathrm{MC}\;(g\;g^{-1}\;\mathrm{DW})=\frac{FW-DW}{DW}$$

### 4.4. Embryo Germinability After Physical Dehydration (Experiment 2)

In this study, 10 healthy CAIRNS embryos were subjected to physical dehydration, with their moisture content measured at 0, 5, 6, and 7 h (time points selected from Experiment 1). Additional lots of 20 dehydrated embryos were used to assess viability at the same htime intervals. Viability was determined by individually transferring the embryos to culture vessels (3 × 15 cm; diameter/height) containing 10 mL of solidified (Gelzan 3 g L^−1^, pH 5.7) Y3 germination medium. The embryos were incubated in the dark for ca. 4 weeks and at the first signs of germination the culture vessels were transferred to a culture room maintained at 25 ± 1 °C, under a 12/12-h (day/night) photoperiod using white, fluorescent light (200 μmol m^−2^ s^−1^). Germination progress was monitored weekly for 12 weeks. Successful germination (defined as the emergence of a healthy shoot measuring 1.5 cm or more) was taken as the measure of viability.

### 4.5. Embryo Size on Germinability After Physical Dehydration (Experiment 3)

In this study 10 large (ca. 8 × 5 mm in height/diameter; weighing 800 to 900 mg FW) and 10 small (ca. 5 × 4 mm in height/diameter; weighing 500 to 550 mg FW) healthy CAIRNS embryos were physically dehydrated, with their moisture content determined after 0, 4, 5, and 6 h of dehydration. In further lots of 20 large and small dehydrated embryos viability assessments were made after 0, 4, 5, and 6 h of dehydration. Viability was assessed as previously described (Section 2.4).

### 4.6. Sucrose Pre-Treatments on Tissue Dehydration and Embryo Viability (Experiment 4)

In this study, 10 large (>700 mg FW), healthy NYD embryos were used for each experimental setup. The embryos were pre-cultured on a solid Y3 medium supplemented with sucrose at concentrations of 0.2, 0.3, 0.4, or 0.5 M for 3, 5, or 7 days. The culture vessels were placed into a culture room maintained at 25 ± 1 °C, under a 12/12-h (day/night) photoperiod using white, fluorescent light (200 μmol m^−2^ s^−1^). Following the pre-treatment phase, the embryos were carefully dried on sterile filter paper and subjected to rapid physical dehydration for durations ranging from 0 to 6 h. Embryos viability assessments were made after each dehydration time.

### 4.7. Experimental Design and Statistical Analysis

For Experiment 1, there were 10 embryos from each of the two varieties and each embryo was regarded as a replication within that treatment. The data obtained from the two varieties was pooled as there were no significant differences discovered between the two varieties. For Experiments 2, 3 and 4 the moisture content data was gathered from 10 replications each consisting of one embryo, while viability was determined from three replicates of 20 embryo for experiment 2 and 3 and three replications of 10 embryo for experiment 4 at each of the dehydration times. All datasets, including both moisture content and germination data, were subjected to statistical analysis for variance using Analysis of Variance (ANOVA). Mean values were subsequently compared using Fisher’s Least Significant Difference (LSD) test, employing the Statistical Analysis System (SAS; Version 9.4). Due to the germination data not meeting the assumptions of normality and equal variance, a square root transformation was applied before conducting the ANOVA.

## 5. Conclusions

This study outlines the functionality of a newly developed desiccator, capable of rapidly, physically dehydrating coconut embryos while retaining their viability. This is an initial and important step in the conservation of germplasm by cryopreservation. Comparative analysis with a previously constructed apparatus demonstrated superior performance in terms of drying efficiency, with the new apparatus achieving a 2-h faster attainment of 20% FW, or a 25% faster rate as compared to the previous method [10]. Further enhancement to drying speed were realized, reducing the time to reach 20% FW to 4 h through the utilization of a sucrose pre-treatment. However, the impact of this treatment on post-cryopreservation germination requires further investigation. Additionally, small embryos exhibit lower tolerance to rapid, physical dehydration as compared to their larger counterparts, underscoring the significance of selecting robust, large embryos for the establishment of a viable cryopreservation repository for coconut germplasm. In conclusion, the devised rapid dehydration apparatus in conjunction with sucrose pre-treatment represents a promising avenue for enhancing the dehydration of coconut embryos, a critical facet in refining the cryopreservation protocol.

## Data Availability

Data are contained within the article.
